# PReS-FINAL-2008: Juvenile dermatomyositis: a report of 22 cases

**DOI:** 10.1186/1546-0096-11-S2-P21

**Published:** 2013-12-05

**Authors:** A Souto, N Alvarez, A Barros, L Fernandez, B Lopez, I Calvo

**Affiliations:** 1 Hospital Clinico Santiago Compostela, Santiago de Compostela, Spain; 2 Hospital Universitario A Coruña, A Coruña, Spain; 3 Hospital La Fe Valencia, Valencia, Spain; 4 Hospital La Fe Valencia, Santiago de Compostela, Spain

## Introduction

Juvenile dermatomyositis is a chronic inflammatory muscular disease that affects every year 1.5-3 children permillion and also produces systemic, skin, joint, digestive and respiratory manifestations.

## Objectives

To describe the main epidemiological, clinical andanalytical aspects of Juvenile dermatomyositis diagnosed in La Fe Hospital (Valencia, Spain).

## Methods

Patients diagnosed of Juvenile dermatomyositis in La Fe Hospital (Valencia, Spain) since June 1992 to March 2013. Dates are shown in median and interquartile range for non-parametric variables, and for parametric variables is given as mean and standard deviation. To assess response to treatment, we used Wilcoxon test for paired measures of non-parametric variables and t-student for parametric variables.

## Results

We included 22 patients, 45.5% women, age at diagnosis 7.6 (3.7-10.8) years and evolution time 2.75 (1-5) months.

The most frequent initial manifestation were muscle weakness (40%) and skin alterations (40%). Also constitutional symptoms (15%) and myalgia (5%). They presented muscle weakness (100%), heliotrope rash (100%), Gottron papules (90.9%), calcinosis (27.3%), other skin lesions such as telangiectasias, oral ulcers, livedo reticularis and purpura (55.5%), mialgia (55.5%), arthralgia (55.5%), arthritis (50%), constitutional symptoms (50%), esophageal involvement (31.8%), gastrointestinal (28.6%), Raynaud (18.2%), fever (27.3%), anasarca (18.2%), dysphonia (18.2%), and lung disease (18.2%).

Patients were treated with corticosteroids, immunoglobulins and immunosuppressants such as methotrexate, tacrolimus, cyclosporine and antimalarial, alone or in combination, with significant improvement.

CPK at diagnosis was 1114 UI/I (280-4952), and 73 (46-106) after 3 months, with significant improvement (p = 0.0010). Aldolase at diagnosis 15.45 U/L (12-37) and 6 (4-10)after 3 months (p = 0.0148). GOT at diagnosis 149 U/L (73-180) and 28 (25-38) after a year (p = 0.0005). GPT at diagnosis 102 U/L (44-187) and 23 (17-36) after a year (p = 0.0003). ESR at diagnosis 18 mm (16-36.5) and 13 (7-18) after a year (p = 0.0139). Strength at diagnosis was 2.5 ± 1 and 4.25 ± 0.87 after 3 months (p < 0.00001).

Capilarocopy was realised in most of patients at diagnosis and showed pathological capillary patterns (decrease in the number of capillaries, enlargement, megacapillaries, and bleeding), with improvement in subsequent assessments.

## Conclusion

Dermatomyositis is a rare disease of childhood. Children we presented had muscle weakness and heliotrope erythema (100%) and in varying proportions Gottron's papules, calcinosis, Raynaud, artromialgias and general symptoms. We propose a comprehensive patient monitoring, assessing muscle strength and laboratory findings as CPK, aldolase, GOT, GPT and ESR but also by capillaroscopy. Capillaroscopy may be indicative of dermatomyositis outbreak, has diagnosis value and may observe an early improvement pattern when patients are in remission.

## Disclosure of interest

None declared.

